# Behavior of Tilted Angle Shear Connectors

**DOI:** 10.1371/journal.pone.0144288

**Published:** 2015-12-07

**Authors:** Koosha Khorramian, Shervin Maleki, Mahdi Shariati, N. H. Ramli Sulong

**Affiliations:** 1 Department of Civil Engineering, Sharif University of Technology, Tehran, Iran; 2 Department of Civil Engineering, University of Malaya, Kuala Lumpur, Malaysia; Northwestern Polytechnical University, CHINA

## Abstract

According to recent researches, angle shear connectors are appropriate to transfer longitudinal shear forces across the steel-concrete interface. Angle steel profile has been used in different positions as L-shaped or C-shaped shear connectors. The application of angle shear connectors in tilted positions is of interest in this study. This study investigates the behaviour of tilted-shaped angle shear connectors under monotonic loading using experimental push out tests. Eight push-out specimens are tested to investigate the effects of different angle parameters on the ultimate load capacity of connectors. Two different tilted angles of 112.5 and 135 degrees between the angle leg and steel beam are considered. In addition, angle sizes and lengths are varied. Two different failure modes were observed consisting of concrete crushing-splitting and connector fracture. By increasing the size of connector, the maximum load increased for most cases. In general, the 135 degrees tilted angle shear connectors have a higher strength and stiffness than the 112.5 degrees type.

## Introduction

### General

**Fig 1:**
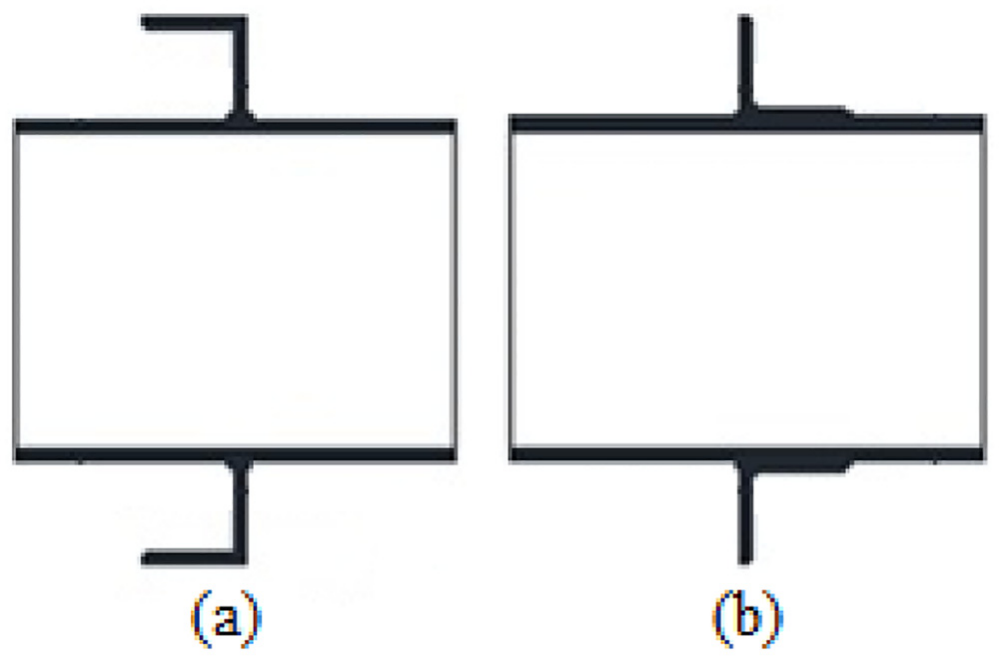
C-shaped angle connectors (a) and L-shaped angle connectors (b).

The use of steel-concrete composite beams in the construction industry has been increasing in the past two decades. The shear connection between the steel beam and the concrete slab in these composite systems plays a vital role in the strength and behavior of these members. Common shear connectors include headed studs, Perfobond ribs, channels and wires. Recently, the demand for innovative shear connectors has led to other forms of connection using steel angles, cold formed shapes and powder actuated attachments. In the developing countries, the stud connector is less popular due to expensiveness of manufacturing and installation and lack of skilled labor. Although headed stud and Perfobond rib connectors are quite appropriate as shear connectors, channel and angle shear connectors have the advantage of being readily available and easily installable. These connectors have a higher load carrying capacity as well. Inspection rules like bending test that are needed for stud connectors are not necessary for these types of shear connectors. Placement of transverse slab reinforcement is not problematic when using these shear connectors as compared to Perfobond connectors. As a result, channel and angle shear connectors are good choices to overcome the restraints and difficulties of using the headed studs and Perfobond shear connectors in composite beams. Angle shear connectors can be used in different shapes (see Fig 1). L-shaped and C-shaped angle connectors have been studied previously.

In this paper, a new idea is investigated by tilting the C-shaped angle position with respect to the vertical axis and observing the effects on the shear strength of the connector. Eight push-out tests are performed to investigate the effects of different angle parameters on the ultimate load capacity of connectors.

### Literature review

There are only a limited number of researches conducted on angle shear connectors in the past. The results of push out tests on several shear connectors including L-shaped angle shear connectors were reported by Rao [1]. The test results showed that C-shaped angle shear connectors provided reasonable flexibility and had reasonable load carrying capacity.

In 1987, the first and foremost applicable work on angle shear connectors was published by Yokota et al. [2]. The study investigated the ultimate strength and deformation of various kinds of shear connectors including C-shaped angles, channels, and T-shaped shear connectors. It was concluded that shapes and directions of shear connectors and concrete strength greatly affect the mode of failure of specimens in the push-out tests. In their research, ultimate strength of connectors was obtained based on 58 push-out test specimens and the following empirical equation was given for the shear strength of C-shaped angle connectors.

$$Q=88w\sqrt{t}\sqrt{f_{c}^{\prime }}$$ (1)

Where:

*Q* = nominal strength of an angle shear connector (kgf)

*t* = web thickness of angle shear connector (cm)

$f_{c}^{\prime }=compressive\;cylinder\;strength\;of\;concrete\;(\frac{kgf}{cm^{2}})$

*w* = length of angle (cm)

In a research by Ciutina and Stratan [3], five different types of shear connectors comprising L-shaped angle shear connectors, were subjected to cyclic and monotonic loading using push-out specimens. It was mainly concluded that cyclic loading makes a reduction of 10–40% in shear resistance of all connectors including L-shaped angle shear connectors when compared to corresponding monotonic loading.

A provision for the design of L-shaped angle shear connectors is provided by the European standard (Eurocode 4) on the design of steel-concrete composite structures as well [4].

There are also some researches on the behavior of C-shaped angle shear connectors. A technical note by Hiroshi and Osamu [5] focused on investigation of the ultimate strength and deformation of different types of shear connectors comprised of C-shaped angles, channels, and T-shaped shear connectors in composite members. It was concluded that shapes and directions of shear connectors and concrete strength had great effects on the mode of failure of push-out specimens. An empirical equation regarding the load-carrying capacity of all shear connectors was suggested in this research.

In a research by Choi et al. [6, 7], fatigue strength of welded joint between C-shaped angle shear connectors and bottom plate in steel-concrete composite slabs was investigated through fatigue tests and finite element analyses. The research confirmed that the stress level at the welded joint was low and much lesser than the fatigue limit.

Fukazawa et al. [8] carried out a wheel truck test on composite slab using C-shaped angle shear connectors to clarify the performance and the applicability to continuous composite steel girder bridges under moving load conditions. Based on the results, they concluded that the composite slab has sufficient fatigue durability and stiffness.

Saidi et al. [9] investigated the relationship between transferred shear force and relative displacement on C-shaped angle and T-shaped shear connectors used in a steel-concrete sandwich beam, and they developed a numerical model.

Ros and Shima [10] developed a new beam type test method to examine the shear force-slip relationship of C-shaped angle shear connectors. The experimental results revealed that the direction of shear force on shear connector has an effect on the shear capacity of shear connectors.

A finite element model was developed using ABAQUS [11] software and validated with push-out laboratory tests by Khalilian [12]. She suggested a new equation to predict the shear strength of C-shaped angle shear connectors using a parametric study as follows,

$$Q=4300L^{0.64}t^{0.27}f_{c}^{0.11}\leq 0.6F_{u}.t.L$$ (2)

Where:

*Q* = shear strength of angle connector (N)

*t* = thickness of angle (mm)

*L* = angle length (mm)

*F_u_* = ultimate strength of steel (MPa)

*fc* = compressive cylinder strength of concrete (MPa)

In recent researches by Shariati et al. [13–16], the behaviour of C-shaped angle shear connectors in normal and high strength concrete under monotonic and reversed cyclic loading was investigated.

According to the experimental studies on C-shaped shear connectors, consisting of channel and angle shear connectors, which were conducted by Maleki et al. [17–19] as well as by Shariati et al. [13–16], and the similarity of channels and angles (except for one leg), it is concluded that push-out test is appropriate method to find out the load-displacement behavior of tilted angle shear connectors. Thus, in the current research, an extensive study on the behaviour of tilted shaped angle shear connectors under monotonic loading is conducted.

## Test Program

### Materials and mix properties

**Fig 2:**
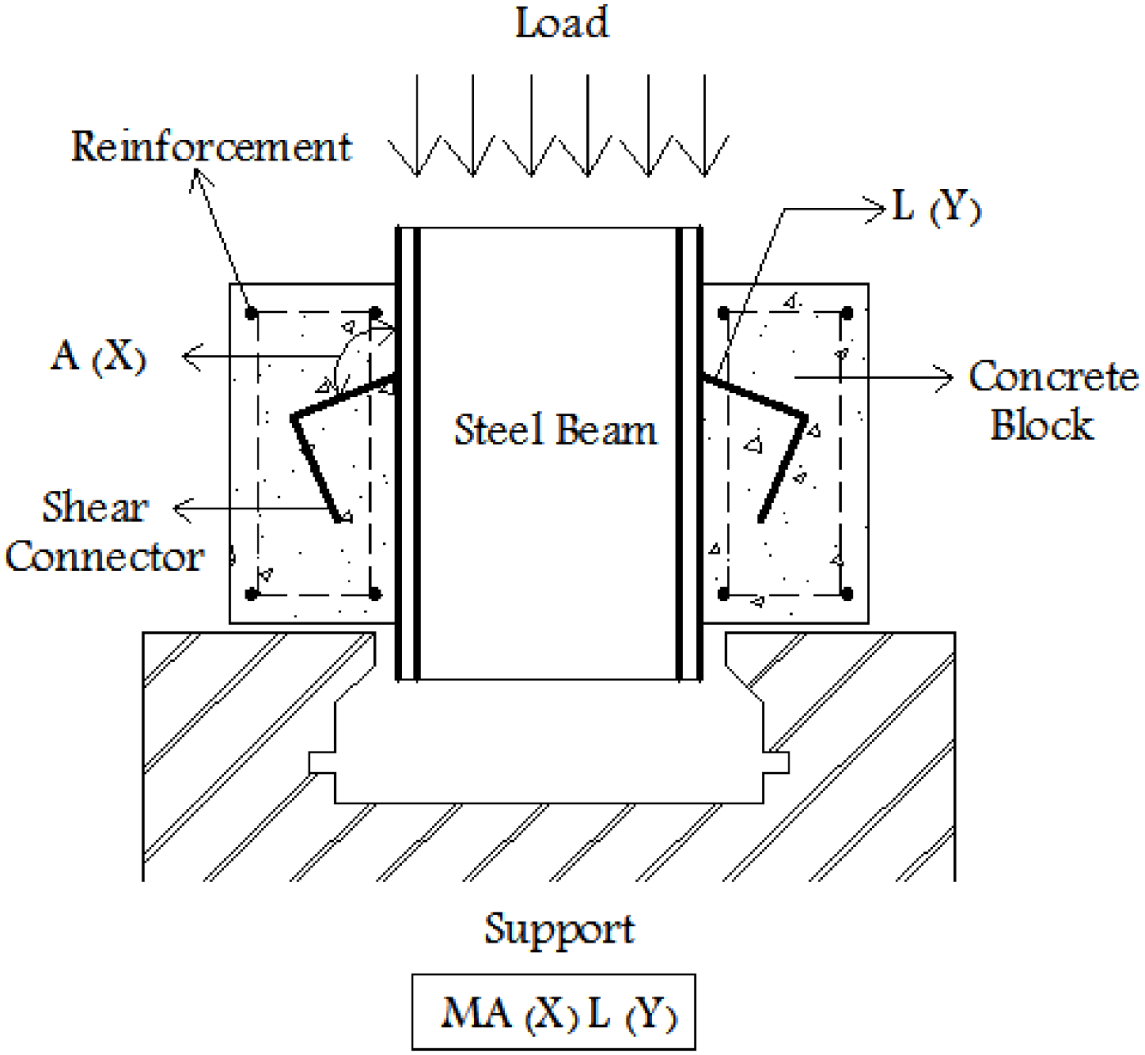
Schematic push-out test setup and symbols for naming.

Details of the concrete mix proportion used in the test specimens are presented in Table 1. The maximum nominal aggregate size of 19 mm was used. The 28 days cylindrical compressive strength of specimens is presented in Table 2. The cement used in all mixes was normal Portland cement, which corresponds to ASTM type II. In all specimens, confining steel bars with nominal diameter of 10 mm and yield stress of 300MPa were used. Three types of tilted-shaped angles consisting of L60, L80, and L100 with leg thicknesses of 6mm, 8mm, and 10 mm, respectively were tested. The tilted angles are 112.5 and 135 degrees measured with respect to the surface of the attached beam (see Fig 2). The angle lengths were 50 mm except for specimens 7 and 8 which had a length of 100mm. Details of tensile test on a typical steel angle are presented in Table 3.

**Table 1:** Mix proportion.

Material	Cement	Water	Sand	Gravel
Weight ratio	1	0.42	2.75	1.75

**Table 2:** Specimens’ description.

No.	Specimen's Name	Length (mm)	Tilted Angle (degrees)	Angle Size	Concrete Strength (MPa)
1	MA 112.5 L60	50	112.5	L60x60x6	27.35
2	MA 112.5 L80	50	112.5	L80x80x8	19.44
3	MA 112.5 L100	50	112.5	L100x100x10	26.12
4	MA 135 L60	50	135	L60x60x6	25.48
5	MA 135 L80	50	135	L80x80x8	19.97
6	MA 135 L100	50	135	L100x100x10	31.11
7	MA* 112.5 L80	100	112.5	L80x80x8	25.26
8	MA* 135 L80	100	135	L80x80x8	24.41

**Table 3:** Steel properties used in push-out specimens.

L-Shape's size	F_y_ (MPa)	ε_y_ %	F_u_ (MPa)	ε_u_ %
L60	377.68	0.19	500.73	28.33

### Push-out specimens

**Fig 3:**
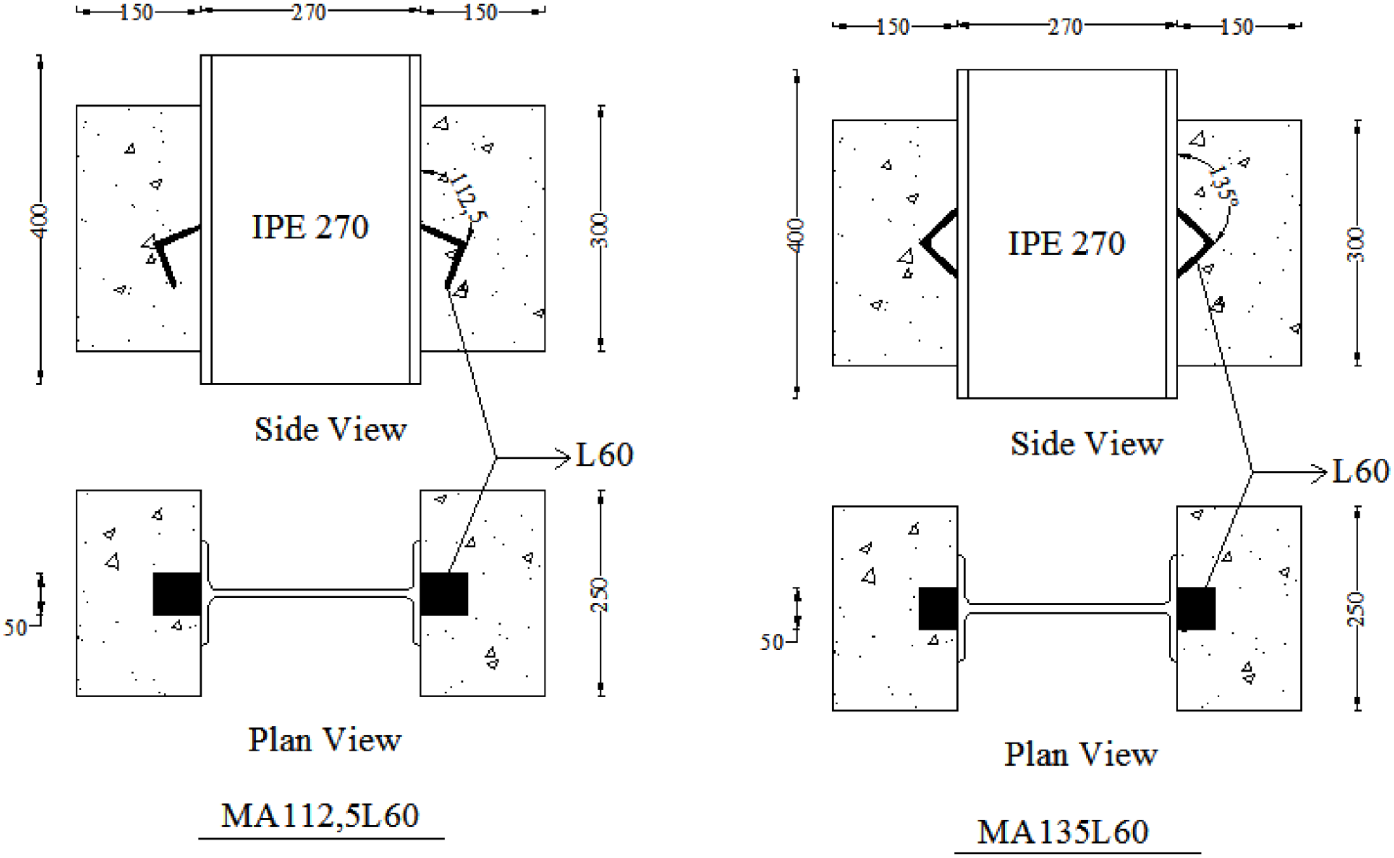
Schematic presentation of specimens.

Push-out specimens consist of a rolled steel IPE270 profile with two tilted-shaped angle shear connectors attached to each flange. All of the specimens are confined with closed rectangular steel stirrups of 10mm diameter at the two sides of concrete block. These tilted-shaped angle shear connectors were embedded in 150x250x300 mm concrete blocks. The angles were fillet welded to the beam on both sides. Note that for MA135 specimens, both legs are welded to the beam. Fig 2 illustrates the schematic test setup and the designation that used for naming the specimens. This includes the letter M for monotonic loading and A (X) for the tilted angle and L (Y) for the angle size. Fig 3 shows the test setup for specimens MA112.5L60 and MA135L60.

### Push-out test

**Fig 4:**
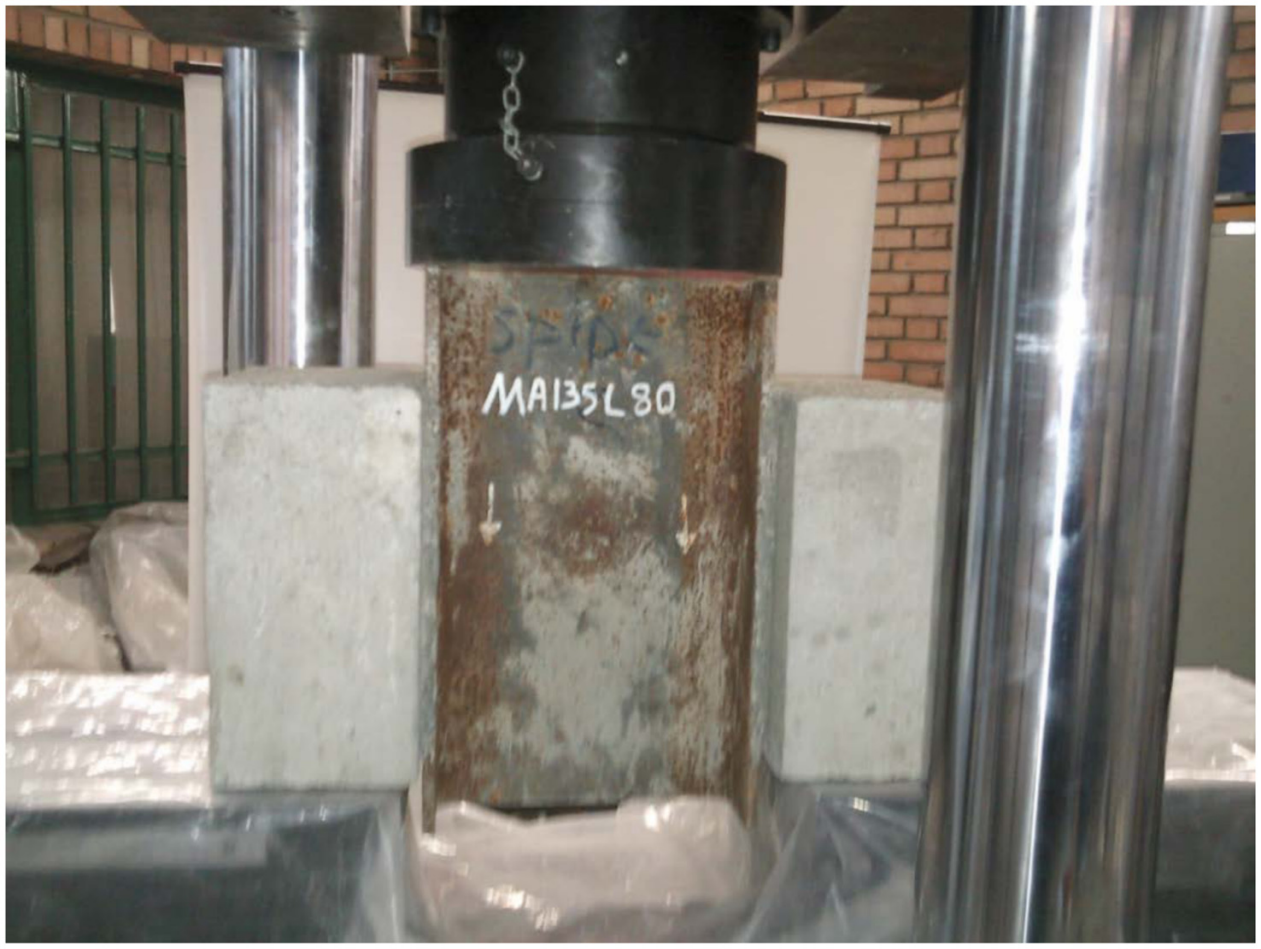
Test setup for MA135L80.

Push-out test specimens were tested by Dartec Universal testing machine with capacity of 1000kN. The loading method was displacement control with a rate of 0.1mm/s. The uniform load was applied on the top of the steel beam while the bottoms of concrete blocks were supported on a rigid base. The actual push-out test setup is shown in Fig 4.

## Test Results and Discussion

### Failure modes

**Fig 5:**
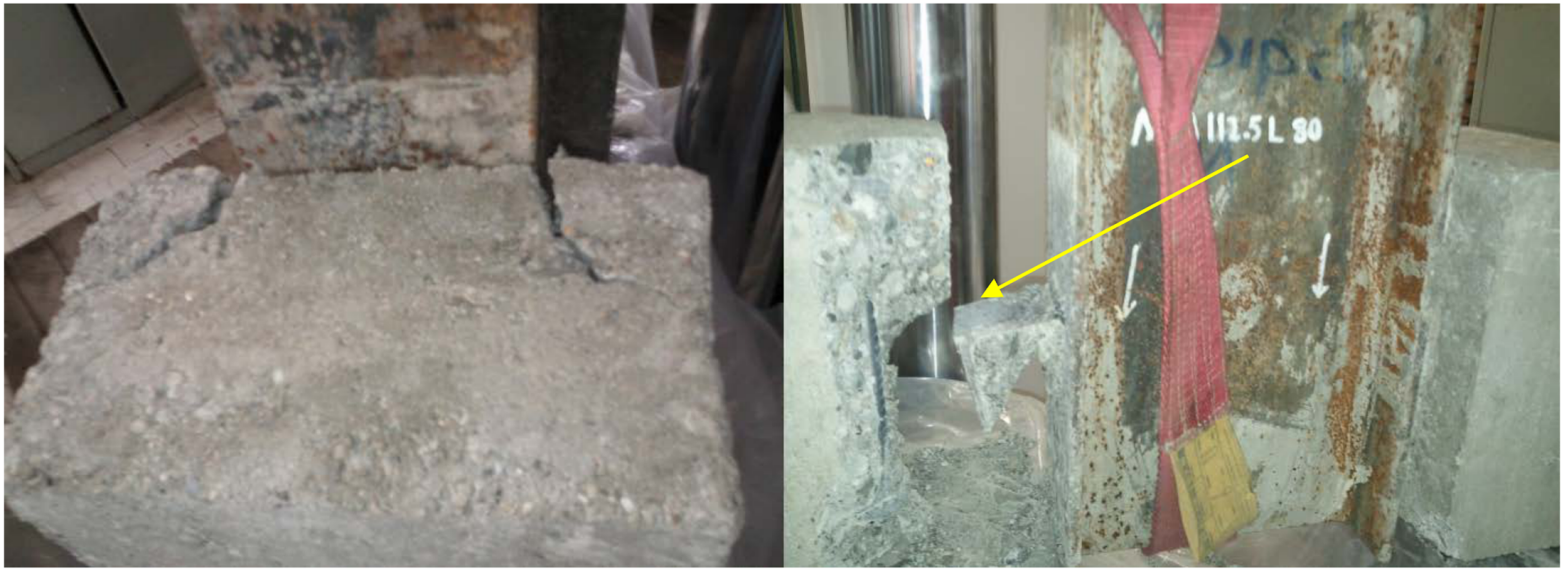
Typical concrete crushing-splitting mode of failure (MA112.5L60).

**Fig 6:**
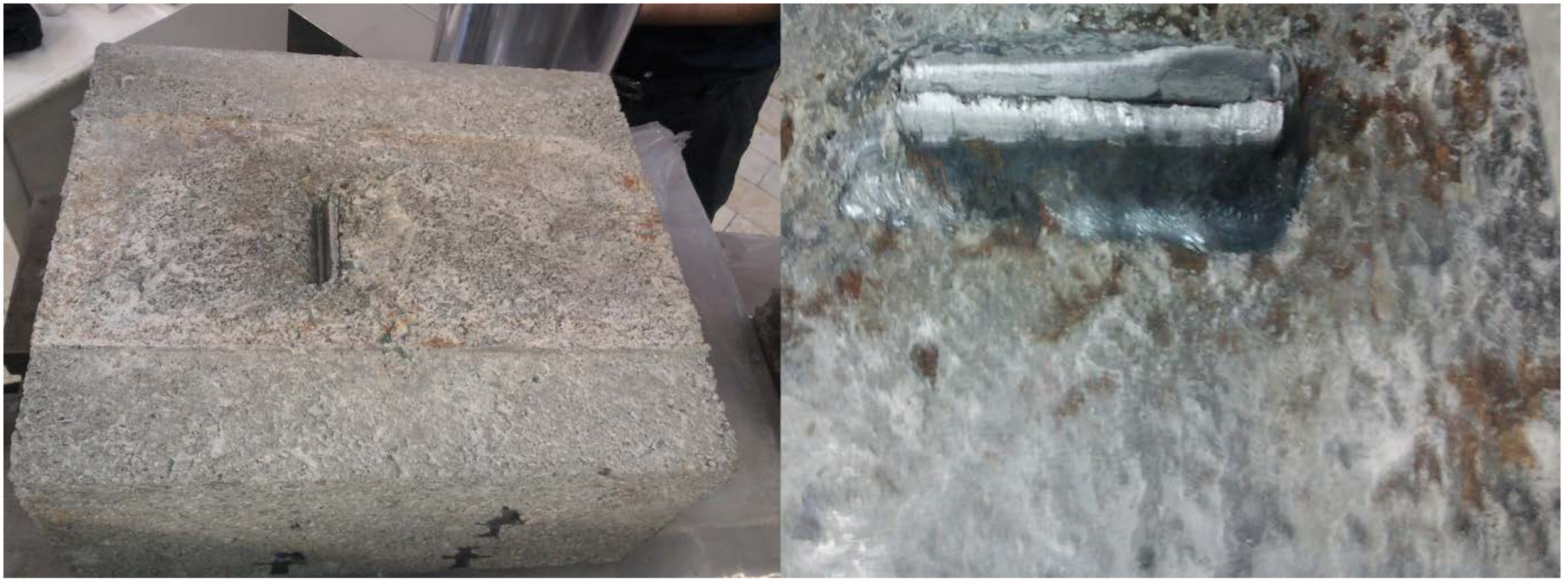
Connector fracture mode (MA112.5L100).

There were two observed modes of failure consisting of concrete crushing-splitting and connector fracture as presented in Figs 5 and 6, respectively. The first mode is a result of high compressive stresses below and tensile stresses above the connector. The second mode starts with yielding of the connected leg just above the weld due to shear and eccentric moment which entails complete fracture. Table 4 shows the modes of failure in all specimens.

**Table 4:** Test results.

No.	Specimen's name	Failure mode	Failure load (kN)
1	MA112.5L60	Concrete crushing-splitting	101.56
2	MA112.5L80	Concrete crushing-splitting	115.4
3	MA112.5L100	Connector fracture	120.09
4	MA135L60	Concrete crushing-splitting	76.36
5	MA135L80	Concrete crushing-splitting	134.11
6	MA135L100	Concrete crushing-splitting	201.13
7	MA*112.5L80	Concrete crushing-splitting	179.04
8	MA*135L80	Concrete crushing-splitting	156.18

It would be useful to mention that the tilted angle shear connector behaves more rigid behaviour compared to most of the stud connectors and can be considered as non ductile connector. The ultimate strength is reached at slips below 2 mm. However, with larger angle size the behaviour becomes more ductile for 112.5 degree tilted connector. For example, MA112.5L100 specimen has a ductile behaviour with over 5 mm of slip before the connector failure.

### Load-displacement behavior

**Fig 7:**
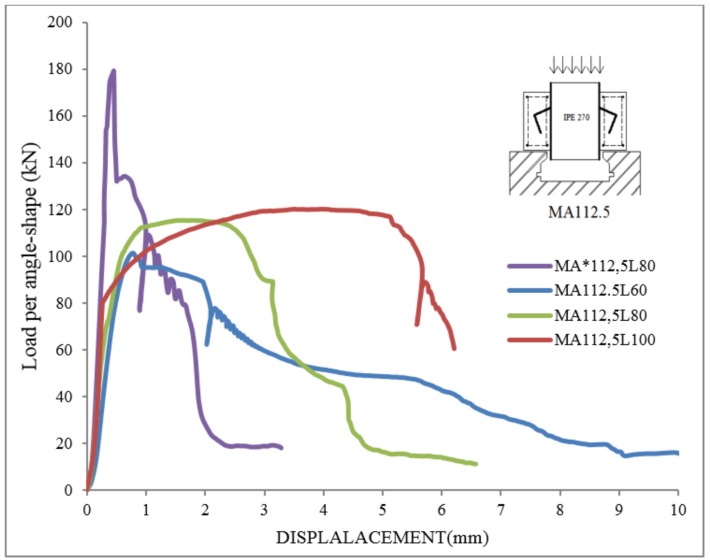
Load-displacement curves in specimens with tilt angle of 112.5.

**Fig 8:**
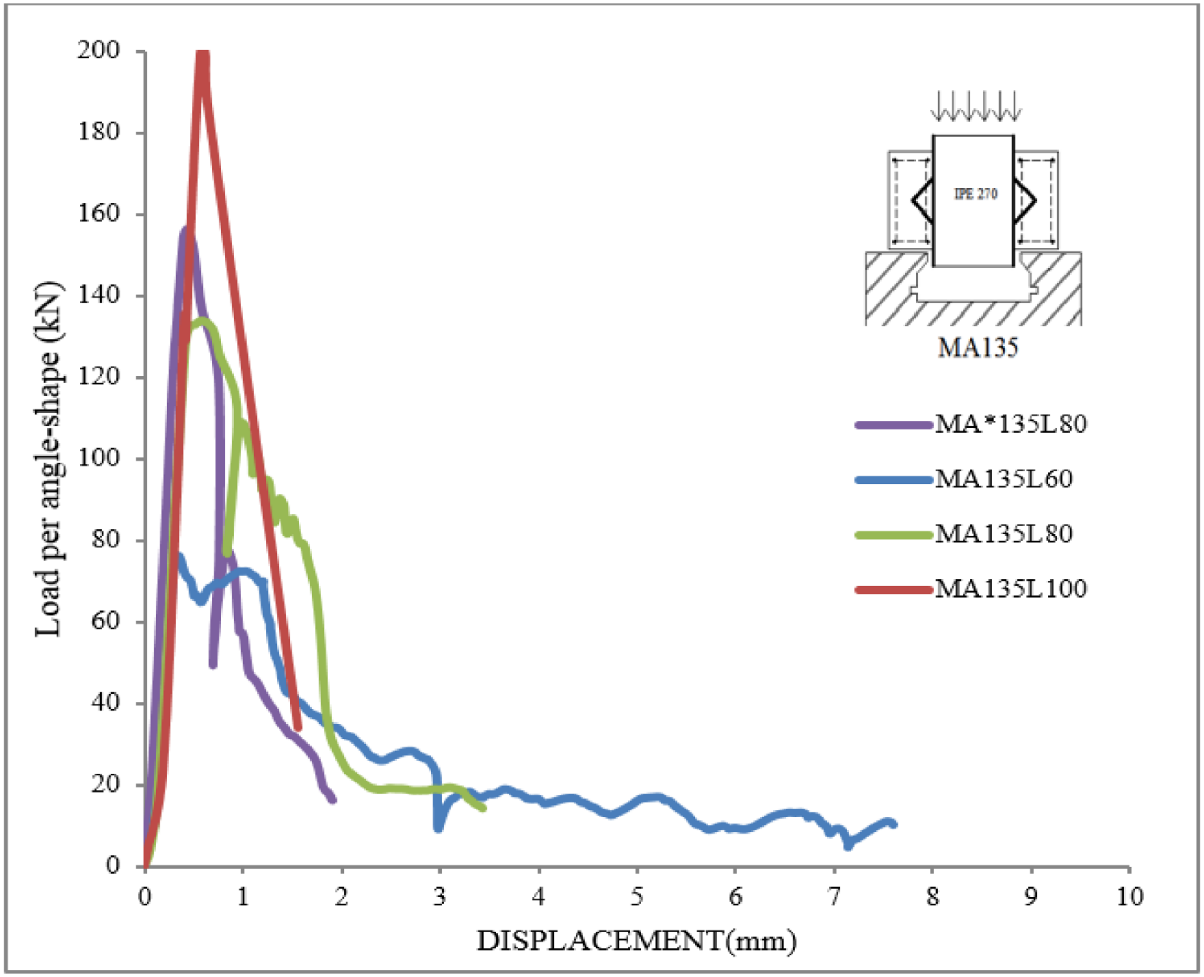
Load-displacement curves in specimens with tilt angle of 135.

In Fig 7 in S1 File and Fig 8, the applied load per connector versus displacement are shown for all specimens with tilted angles of 112.5 and 135 degrees, respectively. The loads and displacements were measured automatically by the Universal testing machine. The failure loads are presented in Table 4.

It is seen that at peak load the relative displacement is around 1 to 6mm for specimens with tilted angle of 112.5, while this value is around 0.5 to 1.0 mm for specimens with tilted angle of 135. In addition, the failure in specimens with tilted angle of 135 occurs abruptly. Therefore, the MA112.5 type specimens were more ductile than MA135. However, in most cases, higher strength can be achieved with MA135 specimens when the angle size is big enough for the concrete to penetrate inside the angle connector. This V shaped space between the beam and the connector in MA135 specimens can confine concrete and increase the shear strength as seen in MA135L100.

### Effect of connectors’ geometry

Considering the specimens with the same length of shear connectors, when the height of MA112.5 specimens was increased from 60 to 100 mm, only about 19% increase of shear strength was observed. Therefore, there is no substantial change in ultimate load capacity for these specimens when the height of the connector varies. On the other hand, for MA135 specimens for the same change of connector size a 163% more shear strength was observed.

Considering next the effect of connectors’ length, the ultimate strength of MA112.5 specimen increased about 55% when the length of the connector was changed from 50 to 100 mm. On the other hand, for MA135 specimens, only about 16% increase in shear strength was observed for the same length increase. Therefore, the length of the connector is not as critical for MA135 specimens.

Generally, by increasing the thickness of connector in both types of specimens, it is seen that the maximum load increases. It can be concluded from strength curves that the MA135 type is stiffer and stronger shear connector than MA112.5 type. However, it has less ductility.

### Comparison with tilted and non tilted angle shear connectors

To compare the tilted angle shear connectors with non tilted angle shear connectors, Table 5 is presented. The results are related to two push-out tests with non tilted angle shear connectors which has been performed by Shariati et al [15] in comparison with results from tilted ones. As it is indicated in the table, angle of 135 degree has the highest capacity in comparison with other cases. When L100 is considered, angle with no tilt has higher shear capacity than tilted angle of 112.5 degree. When L80 is compared with L75, tilting of 112.5 degrees gives higher capacity due to its higher height. It is expected that for the case L80, shear connector with non tilted angle connector endure more shear stresses than tilted angle of 112.5 degree. Thus, in conclusion, the tilted angle of 135 has the highest shear capacity and the non tilted angle shear connector has a higher shear capacity than 112.5 degrees tilted connector.

**Table 5:** Comparison between tilted and non tilted angle shear connectors.

Specimen	F(kN)	h(mm)	L(mm)	f_c_(MPa)
A7550-M [15]	109.6	75	50	28.5
MA112.5L80	115.4	80	50	19.44
MA1135L80	134.11	80	50	19.97
A10050-M [15]	141	100	50	28.5
MA112.5L100	120.09	100	50	26.12
MA1135L100	201.13	100	50	31.11

## Conclusions

In this paper the results of eight push-out tests of tilted angle shear connectors were reported. Tilt angles of 112.5 and 135 degrees with respect to the connected beam flange were considered. The effects of angle length and size were noted. All specimens were tested under monotonic loading to obtain the load-displacement relationship. The failure modes observed in all push-out specimens can be classified into two types: the connector fracture and the concrete crushing-splitting. The connector fracture mode of failure showed a ductile behavior with substantial displacement capacity. The test results show that the only mode of failure in specimens with 135 degrees tilt is concrete crushing-splitting. This is due to both angle legs being welded. In addition, there is a smooth surface in the interface of 135 degrees tilted connectors with concrete which makes concrete sliding over the angle easily possible especially, for small angle sizes.

Generally speaking, in both tilted angle shear connectors, increasing the size of connector results higher capacities due to higher thickness. However, in MA135 connectors the higher angle size confines more concrete beneath the angle which increases the capacity substantially. It was found that the length of connector has a profound effect on the capacity of MA112.5 type and very little effect on MA135 type. It can be concluded that MA135 type connectors are stronger and stiffer with less ductility when compared to MA112.5 type.

## Supporting Information

S1 File Load-displacement curves in specimens with tilt angle of 112.5. (XLSX)
