# Correction: Behavior of Tilted Angle Shear Connectors

**DOI:** 10.1371/journal.pone.0148945

**Published:** 2016-02-05

**Authors:** The PLOS ONE Staff

The Funding section should read as follows: The study presented herein was made possible by the University of Malaya Higher Impact Research (HIR) Grant: UM.C/HIR/MOHE/ENG/57 and University of Malaya Research Grant (UMRG): RP004C-13AET. The authors would like to acknowledge the support.

The publisher apologizes for this error.
